# (2*E*)-3-(4-Chloro­phen­yl)-1-(1*H*-pyrrol-2-yl)prop-2-en-1-one

**DOI:** 10.1107/S1600536808010362

**Published:** 2008-04-18

**Authors:** Mujahid Hussain Bukhari, Hamid Latif Siddiqui, M. Nawaz Tahir, Muhammad Ashraf Chaudhary, Amjid Iqbal

**Affiliations:** a Institute of Chemistry, University of the Punjab, Lahore 54590, Pakistan; b University of Sargodha, Department of Physics, Sargodha, Pakistan; c Department of Chemistry, F.C. University, Lahore 54600, Pakistan

## Abstract

In the mol­ecule of the title compound, C_13_H_10_ClNO, the benzene and pyrrole rings are oriented at a dihedral angle of 7.37 (12)°. In the crystal structure, inter­molecular N—H⋯O hydrogen bonds link the mol­ecules into centrosymmetric *R*_2_^2^(10) dimers. There are C—H⋯π inter­actions between benzene and pyrrole rings and a benzene C—H group. A weak π–π inter­action between the pyrrole rings [centroid–centroid distance 3.8515 (11) Å] further stabilizes the structure. There is also a π inter­action between the pyrrole ring and the carbonyl group, with a carbon–centroid distance of 3.4825 (18) Å.

## Related literature

For general background, see: Varga *et al.* (2003 ▶); Katritzky & Rees (1984 ▶); Wu *et al.* (2003 ▶); Nam *et al.* (2003 ▶); Sondhi *et al.* (2005 ▶); Miyazaki *et al.* (2005 ▶). For related literature, see: Powers *et al.* (1998 ▶); Hu *et al.* (2006 ▶); Wang *et al.* (2005 ▶); Zeng & Cen (2006 ▶). For ring motif details, see: Bernstein *et al.* (1995 ▶).
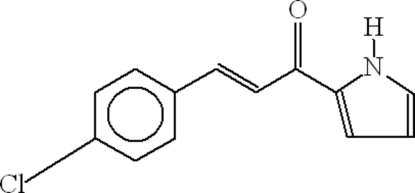


## Experimental

### 

#### Crystal data

C_13_H_10_ClNO
*M* *_r_* = 231.67
Monoclinic, 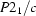

*a* = 13.0401 (7) Å
*b* = 5.6326 (3) Å
*c* = 15.6857 (8) Å
β = 94.979 (3)°
*V* = 1147.76 (10) Å^3^
*Z* = 4
Mo *K*α radiation
μ = 0.31 mm^−1^
*T* = 296 (2) K
0.30 × 0.22 × 0.20 mm

#### Data collection

Bruker Kappa APEXII CCD diffractometer
Absorption correction: multi-scan (*SADABS*; Bruker, 2005 ▶) *T*_min_ = 0.903, *T*_max_ = 0.935
13745 measured reflections
3081 independent reflections
2180 reflections with *I* > 2σ(*I*)
*R*_int_ = 0.026

#### Refinement

*R*[*F* ^2^ > 2σ(*F* ^2^)] = 0.043
*wR*(*F* ^2^) = 0.128
*S* = 1.02
3081 reflections
185 parameters
All H-atom parameters refined
Δρ_max_ = 0.29 e Å^−3^
Δρ_min_ = −0.30 e Å^−3^

### 

Data collection: *APEX2* (Bruker, 2007 ▶); cell refinement: *APEX2*; data reduction: *SAINT* (Bruker, 2007 ▶); program(s) used to solve structure: *SHELXS97* (Sheldrick, 2008 ▶); program(s) used to refine structure: *SHELXL97* (Sheldrick, 2008 ▶); molecular graphics: *ORTEP-3 for Windows* (Farrugia, 1997 ▶) and *PLATON* (Spek, 2003 ▶); software used to prepare material for publication: *WinGX* (Farrugia, 1999 ▶) and *PLATON*.

## Supplementary Material

Crystal structure: contains datablocks global, I. DOI: 10.1107/S1600536808010362/hk2452sup1.cif

Structure factors: contains datablocks I. DOI: 10.1107/S1600536808010362/hk2452Isup2.hkl

Additional supplementary materials:  crystallographic information; 3D view; checkCIF report

## Figures and Tables

**Table 1:** Hydrogen-bond geometry (Å, °)

*D*—H⋯*A*	*D*—H	H⋯*A*	*D*⋯*A*	*D*—H⋯*A*
N1—H1⋯O1^i^	0.83 (2)	2.12 (2)	2.902 (2)	156 (2)
C3—H3⋯*CgA*^ii^	0.938 (18)	2.897 (17)	3.6339 (19)	136.4 (13)
C6—H6⋯*CgB*^iii^	0.94 (2)	2.651 (19)	3.4017 (19)	137.8 (16)

Symmetry codes: (i) 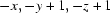; (ii) 
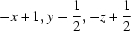
; (iii) 
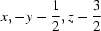
. *CgA* and *CgB* are the centroids of the C1–C6 and N1/C10–C13 rings, respectively.

**Fig. 1.**
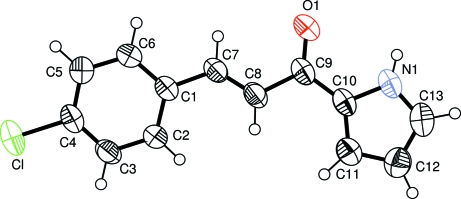
The molecular structure of the title molecule, with the atom-numbering scheme. Displacement ellipsoids are drawn at the 50% probability level.

**Fig. 2.**
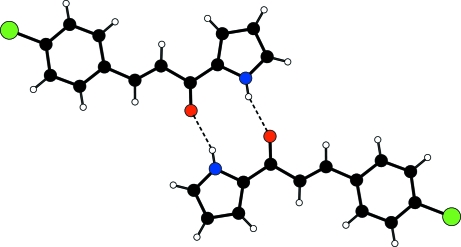
A partial packing diagram of (I), showing the formation of centro- symmetric R22(10) ring motifs. Hydrogen bonds are shown as dashed lines.

## supplementary crystallographic information

### Comment

Chalcones are 1,3-diaryl α,β-unsaturated compounds commonly used as starting materials for the synthesis of several biologically active compounds like pyrimidines and imidazoles (Varga *et al.*,  2003). Most of the chalcones and their derivatives show biological activities such as antimicrobial (Katritzky & Rees, 1984), anti-AIDS (Wu *et al.*,  2003), antimalarial (Nam *et al.*,  2003), anti-inflammatory and analgesic (Sondhi *et al.*,  2005) and antitumor (Miyazaki *et al.*,  2005). While synthesizing different pyrimidine based compounds, we prepared the fine crystals of the title compound, (I), with known method (Powers *et al.*,  1998). We report herein its crystal structure.

The crystal structures of 3-(4-chlorophenyl)-1-(3,4-dimethyl-2,5-dihydro-1H- pyrrol-1-yl)prop-2-enone, (II) (Hu *et al.*,  2006), methyl-3-(1H-pyrrol-2-yl- carboxamido)propionate, (III) (Zeng & Cen, 2006) and 1,3-bis(4-chlorophenyl)- prop-2-en-1-one, (IV) (Wang *et al.*,  2005) have been reported, previously. The title compound, (I), contains the moieties involved in these reported structures.

In the molecule of (I), (Fig. 1), the bond lengths N1-C10 [1.3711 (19) Å], N1-C13 [1.338 (3) Å] and C11-C12 [1.388 (3) Å] are reported as 1.457 (5), 1.461 (5) and 1.328 (6) Å in (II) and 1.369 (2), 1.349 (3) and 1.398 (3) Å in (III), respectively. Rings A (C1-C6) and B (N1/C10-C13) are, of course, planar and they are oriented at a dihedral angle of 7.37 (12)°. So, they are nearly coplanar. The planar central moiety (O1/C7-C9) is oriented with respect to rings A and B at dihedral angles of 8.92 (12)° and 1.94 (15)°, respectively.

In the crystal structure, intermolecular N-H···O hydrogen bonds (Table 1) link the molecules into centrosymmetric R_2_^2^(10) dimers (Fig. 2) (Bernstein *et al.*,  1995), in which they may be effective in the stabilization of the structure. The C—H···π interactions (Table 1) and π—π interactions between B rings CgB···CgB^iv^ [symmetry code: (iv) -x, -y, 1 - z] further stabilize the structure, with a centroid-centroid distance of 3.8515 (11) Å. There is also a π interaction between the ring B at -x, -y, 1 - z and the carbonyl moiety, with C9-centroid distance of 3.4825 (18) Å.

### Experimental

For the preparation of the title compound, a mixture of 4-chlorobenzaldehyde (1.4 g, 10 mmol) and 2-acetyl pyrrole (1.09 g, 10 mmol) was added to MeOH (20 ml) and stirred for 10 min at room temperature. Then, aqueous NaOH solution (10%, 4 ml) was added dropwise with continuous stirring at ambient temperature for 30 min. Light yellow precipitates appeared, to which cold water (40 ml) was added. Yellow colored powder was obtained from the filtrate, which was washed with cold MeOH, and then dried. The residue was recrystallized by dissolving in CHCl_3_ (10 ml) and adding n-hexane dropwise. Fine yellow crystals were obtained (yield; 1.7 g, 73%, m.p. 420-422 K).

### Refinement

H atoms were located in a difference syntheses and refined [N-H = 0.83 (2) Å and U_iso_(H) = 0.073 (6) Å^2^; C-H = 0.937 (19)-0.98 (2) Å and U_iso_(H) = 0.062 (5)-0.091 (7) Å^2^].

### Crystal data

**Table:**

C_13_H_10_ClNO	*F*_000_ = 480
*M**_r_* = 231.67	*D*_x_ = 1.341 Mg m^−^^3^
Monoclinic, *P*2_1_/*c*	Mo *K*α radiation λ = 0.71073 Å
Hall symbol: -P 2ybc	Cell parameters from 2883 reflections
*a* = 13.0401 (7) Å	θ = 2.4–28.7º
*b* = 5.6326 (3) Å	µ = 0.31 mm^−^^1^
*c* = 15.6857 (8) Å	*T* = 296 (2) K
β = 94.979 (3)º	Prismatic, light yellow
*V* = 1147.76 (10) Å^3^	0.30 × 0.22 × 0.20 mm
*Z* = 4	

### Data collection

**Table:**

Bruker KappaAPEXII CCD diffractometer	3081 independent reflections
Radiation source: fine-focus sealed tube	2180 reflections with *I* > 2σ(*I*)
Monochromator: graphite	*R*_int_ = 0.026
Detector resolution: 7.30 pixels mm^-1^	θ_max_ = 29.1º
*T* = 296(2) K	θ_min_ = 1.6º
ω scans	*h* = −17→17
Absorption correction: multi-scan(SADABS; Bruker, 2005)	*k* = −7→7
*T*_min_ = 0.903, *T*_max_ = 0.935	*l* = −21→21
13745 measured reflections	

### Refinement

**Table:**

Refinement on *F*^2^	Secondary atom site location: difference Fourier map
Least-squares matrix: full	Hydrogen site location: inferred from neighbouring sites
*R*[*F*^2^ > 2σ(*F*^2^)] = 0.043	All H-atom parameters refined
*wR*(*F*^2^) = 0.128	*w* = 1/[σ^2^(*F*_o_^2^) + (0.0533*P*)^2^ + 0.3294*P*] where *P* = (*F*_o_^2^ + 2*F*_c_^2^)/3
*S* = 1.02	(Δ/σ)_max_ < 0.001
3081 reflections	Δρ_max_ = 0.29 e Å^−^^3^
185 parameters	Δρ_min_ = −0.30 e Å^−^^3^
Primary atom site location: structure-invariant direct methods	Extinction correction: none

### Special details

**Table:**

Geometry. All e.s.d.'s (except the e.s.d. in the dihedral angle between two l.s. planes) are estimated using the full covariance matrix. The cell e.s.d.'s are taken into account individually in the estimation of e.s.d.'s in distances, angles and torsion angles; correlations between e.s.d.'s in cell parameters are only used when they are defined by crystal symmetry. An approximate (isotropic) treatment of cell e.s.d.'s is used for estimating e.s.d.'s involving l.s. planes.
Refinement. Refinement of F^2^ against ALL reflections. The weighted R-factor wR and goodness of fit S are based on F^2^, conventional R-factors R are based on F, with F set to zero for negative F^2^. The threshold expression of F^2^ > 2sigma(F^2^) is used only for calculating R-factors(gt) etc. and is not relevant to the choice of reflections for refinement. R-factors based on F^2^ are statistically about twice as large as those based on F, and R- factors based on ALL data will be even larger.

### Fractional atomic coordinates and isotropic or equivalent isotropic displacement parameters (Å^2^)

**Table:**

	*x*	*y*	*z*	*U*_iso_*/*U*_eq_	
Cl	0.47349 (5)	−0.30082 (12)	0.06812 (4)	0.0972 (3)	
O1	0.10949 (10)	0.4232 (2)	0.43283 (8)	0.0672 (4)	
N1	0.05564 (11)	0.2306 (3)	0.58749 (10)	0.0572 (4)	
C1	0.29765 (11)	0.0517 (3)	0.26744 (10)	0.0464 (3)	
C2	0.34920 (13)	−0.1623 (3)	0.28485 (11)	0.0525 (4)	
C3	0.40411 (13)	−0.2692 (3)	0.22394 (12)	0.0566 (4)	
C4	0.40726 (13)	−0.1631 (3)	0.14559 (11)	0.0563 (4)	
C5	0.35867 (14)	0.0498 (3)	0.12645 (11)	0.0588 (4)	
C6	0.30445 (13)	0.1551 (3)	0.18805 (11)	0.0537 (4)	
C7	0.23600 (12)	0.1706 (3)	0.32855 (10)	0.0503 (4)	
C8	0.20870 (13)	0.0873 (3)	0.40137 (11)	0.0548 (4)	
C9	0.14204 (12)	0.2253 (3)	0.45499 (11)	0.0522 (4)	
C10	0.11764 (11)	0.1179 (3)	0.53393 (10)	0.0501 (4)	
C11	0.14707 (13)	−0.0945 (3)	0.57282 (11)	0.0566 (4)	
C12	0.10275 (15)	−0.1076 (4)	0.65000 (13)	0.0659 (5)	
C13	0.04627 (14)	0.0958 (4)	0.65667 (12)	0.0668 (5)	
H2	0.3481 (14)	−0.236 (3)	0.3387 (13)	0.066 (5)*	
H3	0.4387 (13)	−0.413 (3)	0.2358 (11)	0.062 (5)*	
H6	0.2713 (14)	0.300 (4)	0.1763 (12)	0.063 (5)*	
H5	0.3607 (15)	0.124 (4)	0.0702 (14)	0.075 (6)*	
H7	0.2116 (14)	0.324 (4)	0.3102 (12)	0.067 (5)*	
H8	0.2289 (15)	−0.069 (4)	0.4203 (13)	0.076 (6)*	
H1	0.0220 (17)	0.353 (4)	0.5749 (14)	0.073 (6)*	
H11	0.1896 (14)	−0.211 (3)	0.5521 (12)	0.063 (5)*	
H12	0.1118 (15)	−0.226 (4)	0.6931 (14)	0.075 (6)*	
H13	0.0089 (17)	0.150 (4)	0.7011 (15)	0.091 (7)*	

### Atomic displacement parameters (Å^2^)

**Table:**

	*U*^11^	*U*^22^	*U*^33^	*U*^12^	*U*^13^	*U*^23^
Cl	0.1189 (5)	0.0979 (5)	0.0810 (4)	0.0298 (3)	0.0442 (3)	−0.0182 (3)
O1	0.0738 (8)	0.0584 (7)	0.0716 (8)	0.0223 (6)	0.0185 (6)	−0.0044 (6)
N1	0.0521 (7)	0.0644 (9)	0.0560 (8)	0.0095 (7)	0.0107 (6)	−0.0144 (7)
C1	0.0443 (7)	0.0442 (7)	0.0509 (8)	0.0037 (6)	0.0063 (6)	−0.0076 (6)
C2	0.0576 (9)	0.0474 (8)	0.0535 (9)	0.0092 (7)	0.0107 (7)	0.0012 (7)
C3	0.0576 (9)	0.0457 (8)	0.0676 (11)	0.0115 (7)	0.0123 (8)	−0.0056 (7)
C4	0.0568 (9)	0.0561 (9)	0.0578 (9)	0.0044 (7)	0.0144 (7)	−0.0152 (7)
C5	0.0653 (10)	0.0612 (10)	0.0512 (9)	0.0050 (8)	0.0120 (7)	−0.0006 (8)
C6	0.0584 (9)	0.0467 (8)	0.0567 (9)	0.0107 (7)	0.0083 (7)	0.0008 (7)
C7	0.0500 (8)	0.0475 (8)	0.0536 (9)	0.0098 (6)	0.0062 (6)	−0.0073 (7)
C8	0.0558 (9)	0.0531 (9)	0.0565 (9)	0.0132 (7)	0.0113 (7)	−0.0078 (7)
C9	0.0481 (8)	0.0550 (9)	0.0537 (9)	0.0078 (7)	0.0062 (6)	−0.0136 (7)
C10	0.0439 (7)	0.0546 (9)	0.0518 (8)	0.0040 (6)	0.0048 (6)	−0.0162 (7)
C11	0.0531 (9)	0.0556 (9)	0.0614 (10)	0.0012 (7)	0.0072 (7)	−0.0107 (8)
C12	0.0647 (10)	0.0701 (12)	0.0638 (11)	−0.0064 (9)	0.0113 (8)	−0.0008 (10)
C13	0.0576 (10)	0.0844 (13)	0.0604 (11)	−0.0042 (9)	0.0163 (8)	−0.0137 (10)

### Geometric parameters (Å, °)

**Table:**

Cl—C4	1.7339 (15)	C5—H5	0.98 (2)
O1—C9	1.232 (2)	C6—H6	0.94 (2)
N1—C13	1.338 (3)	C7—C8	1.312 (2)
N1—C10	1.3711 (19)	C7—H7	0.96 (2)
N1—H1	0.83 (2)	C8—C9	1.481 (2)
C1—C6	1.384 (2)	C8—H8	0.96 (2)
C1—C2	1.396 (2)	C9—C10	1.438 (2)
C1—C7	1.465 (2)	C10—C11	1.382 (2)
C2—C3	1.381 (2)	C11—C12	1.388 (3)
C2—H2	0.94 (2)	C11—H11	0.937 (19)
C3—C4	1.370 (3)	C12—C13	1.371 (3)
C3—H3	0.938 (18)	C12—H12	0.95 (2)
C4—C5	1.377 (2)	C13—H13	0.94 (2)
C5—C6	1.380 (2)		
			
C13—N1—C10	109.55 (16)	C8—C7—C1	127.72 (15)
C13—N1—H1	125.3 (15)	C8—C7—H7	118.5 (12)
C10—N1—H1	124.5 (15)	C1—C7—H7	113.7 (12)
C6—C1—C2	118.16 (14)	C7—C8—C9	121.58 (16)
C6—C1—C7	118.54 (14)	C7—C8—H8	120.7 (12)
C2—C1—C7	123.29 (15)	C9—C8—H8	117.7 (12)
C3—C2—C1	120.73 (16)	O1—C9—C10	121.80 (14)
C3—C2—H2	118.7 (12)	O1—C9—C8	121.29 (16)
C1—C2—H2	120.6 (12)	C10—C9—C8	116.91 (14)
C4—C3—C2	119.22 (16)	N1—C10—C11	106.62 (15)
C4—C3—H3	120.2 (11)	N1—C10—C9	121.27 (15)
C2—C3—H3	120.5 (11)	C11—C10—C9	132.10 (14)
C3—C4—C5	121.75 (15)	C10—C11—C12	108.08 (16)
C3—C4—Cl	119.27 (13)	C10—C11—H11	127.2 (12)
C5—C4—Cl	118.98 (14)	C12—C11—H11	124.7 (12)
C4—C5—C6	118.40 (16)	C13—C12—C11	106.80 (18)
C4—C5—H5	121.3 (12)	C13—C12—H12	124.5 (13)
C6—C5—H5	120.3 (12)	C11—C12—H12	128.6 (13)
C5—C6—C1	121.72 (15)	N1—C13—C12	108.94 (17)
C5—C6—H6	119.6 (12)	N1—C13—H13	120.7 (15)
C1—C6—H6	118.7 (12)	C12—C13—H13	130.3 (15)
			
C6—C1—C2—C3	−0.9 (2)	C7—C8—C9—O1	0.3 (3)
C7—C1—C2—C3	178.38 (16)	C7—C8—C9—C10	−179.47 (15)
C1—C2—C3—C4	−0.3 (3)	C13—N1—C10—C11	−0.09 (19)
C2—C3—C4—C5	1.2 (3)	C13—N1—C10—C9	−179.06 (15)
C2—C3—C4—Cl	−178.39 (13)	O1—C9—C10—N1	0.9 (2)
C3—C4—C5—C6	−1.0 (3)	C8—C9—C10—N1	−179.32 (14)
Cl—C4—C5—C6	178.67 (14)	O1—C9—C10—C11	−177.75 (17)
C4—C5—C6—C1	−0.3 (3)	C8—C9—C10—C11	2.0 (3)
C2—C1—C6—C5	1.2 (3)	N1—C10—C11—C12	−0.27 (19)
C7—C1—C6—C5	−178.12 (15)	C9—C10—C11—C12	178.54 (17)
C6—C1—C7—C8	170.00 (17)	C10—C11—C12—C13	0.5 (2)
C2—C1—C7—C8	−9.3 (3)	C10—N1—C13—C12	0.4 (2)
C1—C7—C8—C9	−177.33 (15)	C11—C12—C13—N1	−0.6 (2)

### Hydrogen-bond geometry (Å, °)

**Table:**

*D*—H···*A*	*D*—H	H···*A*	*D*···*A*	*D*—H···*A*
N1—H1···O1^i^	0.83 (2)	2.12 (2)	2.902 (2)	156 (2)
C3—H3···CgA^ii^	0.938 (18)	2.897 (17)	3.6339 (19)	136.4 (13)
C6—H6···CgB^iii^	0.94 (2)	2.651 (19)	3.4017 (19)	137.8 (16)

Symmetry codes: (i) −*x*, −*y*+1, −*z*+1; (ii) −*x*+1, *y*−1/2, −*z*+1/2; (iii) *x*, −*y*−1/2, *z*−3/2.
